# Normal Uptake of ^11^C-Acetate in Pancreas, Liver, Spleen, and Suprarenal Gland in PET

**DOI:** 10.1155/2017/5478068

**Published:** 2017-09-27

**Authors:** Bogdan Malkowski, Pawel Wareluk, Tomasz Gorycki, Katarzyna Skrobisz, Michal Studniarek

**Affiliations:** ^1^Department of Nuclear Medicine, Oncological Center of Bydgoszcz, Bydgoszcz, Poland; ^2^Department of PET and Molecular Imaging, Collegium Medicum, University of Nicolaus Copernicus, Bydgoszcz, Poland; ^3^Department of Diagnostic Imaging, Medical University of Warsaw, Warsaw, Poland; ^4^Department of Radiology, Medical University of Gdańsk, Gdańsk, Poland

## Abstract

**Purpose:**

^11^C-Acetate is radiotracer being considered an alternative to ^18^F-fluorodeoxyglucose. Evaluation of ^11^C-acetate biodistribution in human parenchymal organs is described.

**Methods and Materials:**

60 consecutive patients referred to ^11^C-acetate PET CT suspected of renal or prostate cancer relapse with negative results (no recurrent tumor) were included in the study. Acquisition from the base of skull to upper thigh was made 20 min after i.v. injection of 720 MBq of ^11^C-acetate. The distribution was evaluated by measuring the uptake in pancreas (uncinate process and body separately), liver, spleen, and left suprarenal gland. Clinical data of included patients showed no abnormalities in these organs.

**Results:**

Biodistributions of ^11^C-acetate radiotracer were compared in different organs. Standardized uptake values of ^11^C-acetate were significantly higher in pancreatic parenchyma (SUV mean 6,4) than in liver (SUV mean 3,3), spleen (SUV mean 4,5), or suprarenal gland (SUV mean 2,7) tissues. No significant difference was found between pancreatic head (SUV mean 6,4) and body (SUV mean 5,9) uptake. In case of all aforementioned organs, there were no differences either between both sexes or between formerly diagnosed tumors (renal and prostate).

**Conclusions:**

Evaluation of ^11^C-acetate uptake differences in parenchymal organs will allow establishing normal patterns of distribution. High pancreatic uptake may be used in quantitative assessment of organ function in diffuse nonneoplastic pathology.

## 1. Introduction

^11^C labelled acetate was a radiotracer first used for the assessment of myocardial viability over two decades ago [1]. Since then it has been evaluated as a promising tracer, mainly in oncological research, and also as a possible alternative to ^18^F-fluorodeoxyglucose (FDG). However, ^11^C-acetate is not as widely used in clinical practice and investigated as FDG. Relatively short half-life of approximately 20 minutes is one of the reasons for the above. Currently, positron emission tomography (PET) examinations with the use of ^11^C-acetate are performed mostly in the fields of oncology, urology, and cardiology, with reports of unusual, rare tumor findings such as thymoma or cerebellopontine angle schwannoma [2].

The purpose of this study was to evaluate distribution of ^11^C-acetate in human parenchymal organs during whole body PET examination combined with computed tomography (CT). Uptake of radiotracer was measured in pancreas (uncinate process and body separately), liver, spleen, and left suprarenal gland.

## 2. Methods and Materials

60 consecutive patients (22 women, 38 men) referred to ^11^C-acetate PET CT suspected of renal or prostate cancer relapse with negative results (no recurrent tumor) were included in the study. 56 patients had kidney cancer and 7 patients had prostate cancer (three had both of them).

PET/CT study were made using Biograph mCT 128. ^11^C-Acetate was produced in our laboratory using Explora Acetate module according to the manufacturer instruction and GMP standards.

Acquisition was made 20 min after i.v. injection of 720 MBq of acetate. The acquisition from the base of skull to 1/3 upper thigh was performed. The parameters of the acquisition and reconstruction are presented in Table 1.

**Figure 1:**
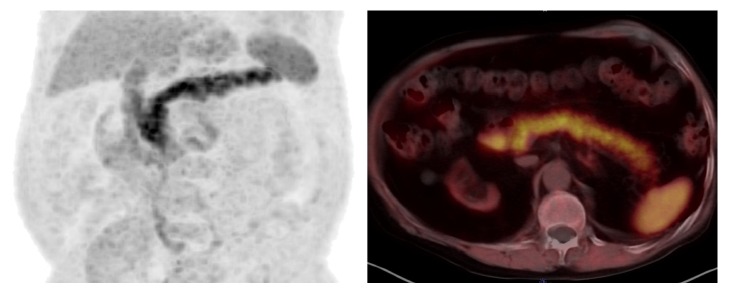
The dominant organ uptake of ^11^C-acetate is seen in the pancreas.

^11^C-Acetate distribution was evaluated by placing the spherical 10 mm VOI and measuring the uptake values with isocontour tool. SUVs (max, peak, and mean) were recorded for pancreas (head and body separately), liver, spleen, and left suprarenal gland (Figure 1). The position of VOIs was the same for all patients and anatomical imagining (CT) was used to allocate them. Right suprarenals were not taken into account because of overlapping radioactivity from the liver. Clinical data of included patients showed no abnormalities in the all organs studied.

Statistical calculations were performed using STATISTICA (ver. 12.0, StatSoft Inc., 2014) statistical package and Excel (Microsoft) spreadsheet. Quantitative variables were characterized by the arithmetic mean, standard deviation, median, minimum and maximum values (range), and 95% CI (confidence interval). In contrast, the qualitative variables were presented using frequencies and percentages. To check whether a quantitative variable came from a normally distributed population, Shapiro-Wilk test was used. Leven (Brown-Forsythe) test was used to test the hypothesis of equal variances. The significance of differences between the two groups (unpaired model) was examined with the following tests: Student's *t*-test (or Welch *t*-test, in the absence of homogeneity of variance) or Mann–Whitney *U* test. The significance of differences between more than two groups was tested by an *F* test (ANOVA) or Kruskal-Wallis test (when ANOVA was inapplicable). If statistically significant differences between groups were present, post hoc tests (Tukey test for *F*, Dunn test for Kruskal-Wallis) were applied. In all the calculations the level of significance was set at *α* = 0.05.

## 3. Results

Of all analyzed organs the head of the pancreas had highest SUVs values (SUV max 9.3, SUV peak 7.9, and SUV mean 6.4). However no statistically significant difference was found between pancreatic head (SUV mean 6,4) and body (SUV mean 5,9) uptake (Table 1). Standardized uptake values of ^11^C-acetate were significantly higher in pancreas (SUV mean of 6,4 for head and 5.9 for body) than in liver (SUV mean 3,3), spleen (SUV mean 4,5), or left suprarenal gland (SUV mean 2,7) (Table 2). In all organs there were no differences either between both sexes or between formerly diagnosed tumors (kidney and/or prostate cancer). The results were presented as an abstract and discussed at ECR 2016 Congress in Vienna [3].

## 4. Discussion

Acetate (CH3COO−) is an ion formed from acetic acid (CH3COOH) by losing hydrogen ion. Physiologically, in human organism acetate is converted into acetyl-CoA and, depending on a cell type, involved in two main different metabolic pathways. The first is tricarboxylic acid cycle, resulting in energy, carbon dioxide, and water. The second, on the contrary, is anabolic pathway, leading to synthesis of cholesterol and fatty acids, which are later incorporated in the form of phospholipids into cell membranes [4]. Both pathways are also important in oncogenesis, as atypical, rapidly dividing cells are in need of energy and substrates for creating cell membranes. On this account the potential intracellular acetate utilization could be monitored in diagnostic and perhaps therapeutic applications in oncology [5]. Until now it was mainly used for imaging of renal, prostate, and bladder cancers.

For diagnostic purposes in nuclear medicine, acetate is labelled with ^11^C carbon isotope produced from ^14^N nitrogen by proton bombardment in a cyclotron. The labelling process requires considerable effort to convert gaseous precursors (radioactive CO2 and methane) into more reactive molecules suitable for reaction with acetate [6]. After that, complete radiotracer is prepared for intravenous injection. Seltzer et al. evaluated ^11^C-acetate estimated absorbed doses for healthy volunteers with pancreas, bowels, liver, kidneys, and spleen getting highest doses [7]. The radiotracer is not excreted in urine under normal circumstances. Few theories exist regarding distribution of ^11^C-acetate, for example, its high concentration in pancreas which may correspond with increased lipid synthesis in acinar cells [7], incorporating into zymogens or generating hydrogen carbonate ions [8]. A recent study showed also potential of evaluating pancreatic exocrine function as ^11^C-acetate activity increased in duodenum after secretin administration [9].

This study's purpose of evaluating ^11^C-acetate uptake pattern in selected abdominal parenchymal organs has been undertaken only a few times before. The results are convergent with previous findings, stating the pancreas as an organ receiving highest absorbed doses [7]. Other organs (liver, spleen, and left suprarenal gland) showed significantly lower uptake of radiotracer, with spleen being second after pancreas. To our knowledge no study up to date tried to evaluate distribution of ^11^C-acetate in different parts of pancreas, based on the fact that the uncinate process and the head have different embryological origins. No statistically significant differences were found in radiotracer uptake in body and uncinate process of pancreas in this study. Inclusion criteria in our study required no previous history of pancreas pathology and normal lab test results, but as other studies show [9, 10] it is possible to use ^11^C-acetate in evaluating pancreas exocrine function.

In some forms of hereditary chronic pancreatitis, that is, coexisting with cystic fibrosis, there are no clinical symptoms of pancreatic exocrine or endocrine insufficiency [11]. The high risk of cancer development in these patients needs more radical treatment, but the patients usually do not accept the proposition until the pancreatic insufficiency is evident. Then ^11^C-acetate PET/CT could be potentially decision-making tool. There is a need to develop more convenient tests to diagnose exocrine pancreatic insufficiency and monitor the disease progression.

The patients included had a history of kidney or/and prostate cancer but there was no difference in radiotracer distribution in both groups, as well as between male and female subjects.

^11^C-Acetate uptake in suprarenal glands seems to be another interesting subject, as it was mentioned in only one study [12] regarding adrenal adenomas. Our results show that suprarenals had lowest uptake of examined organs. However it may be difficult to investigate right suprarenal glands due to problems with overlapping radioactivity from the liver.

## 5. Conclusions

The highest SUV max, SUV mean, and SUV peak values in pancreatic tissue in comparison to liver, spleen, and left suprarenal gland most probably indicate that specific organ function, that is, synthesis of hydrocarbonates or fatty acids, plays significant role in ^11^C-acetate pancreatic uptake. It has to have a diagnostic potential in chronic pancreatic diseases. High pancreatic uptake may be used in the quantitative assessment of organ function in diffuse nonneoplastic pathology. Evaluation of ^11^C- acetate uptake differences in parenchymal organs will allow establishing normal patterns of distribution and should lead to further studies of uptake changes in pancreatic disorders.

## Figures and Tables

**Table 1:** The parameters of acquisition and reconstruction.

	^11^C-Acetate
*CT WB*	
Topogram	Standard
Eff mAs	Care dose 4D
kV	120
Slice	5.0 mm
Acq	32 × 1.2 mm
Pitch	0.8
Direction	Craniocaudal
Kernel	B30f
FoV	780 mm
Increment	3.0 mm

*PET WB*	
Isotope	C-11
Pharm	Acetate
Scan range	Match CT range
Scan duration/bed	2.0 min

*PET recon 1*	
Output image	Corrected
Recon meth	TrueX + tof (UltraHD-PET)
Iteration	2
Subset	21
Image size	200
Filter	Gaussian
Zoom	1.0
FWHM	2.0

*PET recon 2*	
Output image	Uncorrected
Recon meth	Iterative + tof
Iteration	2
Subset	24
Image size	200
Filter	Gaussian
Zoom	1.0
FWHM	2.0
+	Standard AC recon

**Table 2:** Comparison of ^11^C-acetate uptake by organ.

	Pancreas	Pancreas	Liver (*N* = 60)	Spleen (*N* = 60)	Suprarenal gland (*N* = 60)	*P* value
	head	body				
	(*N* = 60)	(*N* = 60)				
*SUV max*						
Mean (SD)	9,3 (2,6)	9,0 (2,8)	5,2 (1,7)	6,4 (1,6)	4,2 (1,8)	0,001
Range	4,6–15,9	4,0–16,1	2,6–9,1	3,5–10,6	2,2–15,3	
Median	9,0	8,4	4,8	6,2	4,0	
95% CI	[8,6; 10,0]	[8,3; 9,7]	[4,7; 5,6]	[5,9; 6,8]	[3,8; 4,7]	

*SUV peak*						
Mean (SD)	7,9 (2,5)	7,2 (2,4)	4,2 (1,4)	5,4 (1,4)	3,5 (1,6)	0,001
Range	3,9–15,0	2,7–13,3	2,1–7,4	2,6–9,2 1,5–13,3		
Median	7,6	7,0	4,1	5,3	3,2	
95% CI	[7,3; 8,5]	[6,6; 7,8]	[3,9; 4,6]	[5,1; 5,8]	[3,0; 3,9]	

*SUV mean*						
Mean (SD)	6,4 (2,0)	5,9 (1,9)	3,3 (1,1)	4,5 (1,2)	2,7 (1,3)	0,001
Range	3,0–12,3	2,3–10,8	1,6–5,7	2,1–7,7 1,4–10,8		
Median	6,2	5,8	3,1	4,3	2,6	
95% CI	[5,9; 6,9]	[5,4; 6,3]	[3,0; 3,6]	[4,2; 4,8]	[2,4; 3,1]	
